# A Histological Conundrum With a Distinctive Biochemical Marker: A Case Report of Primary Pulmonary Choriocarcinoma

**DOI:** 10.7759/cureus.100377

**Published:** 2025-12-29

**Authors:** Timothy Ming Him Yeung, Raymond Yu O, Karen Ka-Wan Yuen, Sidney Tam

**Affiliations:** 1 Division of Chemical Pathology, Department of Pathology, Queen Mary Hospital, Hong Kong, HKG; 2 Department of Pathology, The University of Hong Kong, Hong Kong, HKG; 3 Division of Anatomical Pathology, Department of Pathology, Queen Mary Hospital, Hong Kong, HKG

**Keywords:** gata3, hcg, immunohistochemistry (ihc), primary choriocarcinoma, primary pulmonary choriocarcinoma, sall4, squamous cell carcinoma (scc)

## Abstract

Primary pulmonary choriocarcinoma (PPC) is a rare, malignant germ cell neoplasm with a poor prognosis that is difficult to diagnose. Measurement of human chorionic gonadotropin (hCG), which is secreted by PPCs, facilitates the detection of PPCs.

We describe a 69-year-old man who presented with shortness of breath and was found to have a large lung tumor. Histological examination of the biopsied right lung mass revealed tumor cells with enlarged pleomorphic nuclei, irregular nuclear contour, and conspicuous nucleoli. Immunostaining of the tumor cells was positive for MNF-116 and patchily positive for p40, while negative for leukocyte common antigen (LCA (CD45)), S100, smooth muscle actin (SMA), thyroid transcription factor 1 (TTF-1), and synaptophysin. While such features mimicked squamous cell carcinoma, an extremely elevated serum total hCG level of 24544 IU/L prompted further immunohistochemical staining for choriocarcinomas. The tumor was positive for spalt-like transcription factor 4 (SALL4) and GATA-binding protein 3 (GATA3), which established the diagnosis of PPC.

This case illustrates the histopathological difficulties in diagnosing PPC and the utility of serum hCG measurement in the detection of PPC. Serum hCG measurement is useful as an initial investigation for possible PPC in patients with non-small cell lung tumors, which should be followed by immunohistochemical staining for markers of choriocarcinoma.

## Introduction

Primary pulmonary choriocarcinoma (PPC) is a highly malignant germ cell neoplasm that arises de novo in the lung, which is a tumor of poor prognosis that is often misdiagnosed or missed due to its nonspecific clinical characteristics and rarity [1]. PPC may resemble other histological types [2-6] or intermingle with other tumor types [2,7-9], which increases the difficulty of diagnosis by histopathology alone. Unlike most germ cell tumors, which most often arise in midline structures, the occurrence of PPCs in the lungs often evades early diagnosis. The estimated incidence of primary pulmonary choriocarcinoma is 1 per 12 million individuals per year [10]. The measurement of serum human chorionic gonadotropin (hCG), which is secreted by PPC, would facilitate its identification [1].

## Case presentation

**Figure 1:**
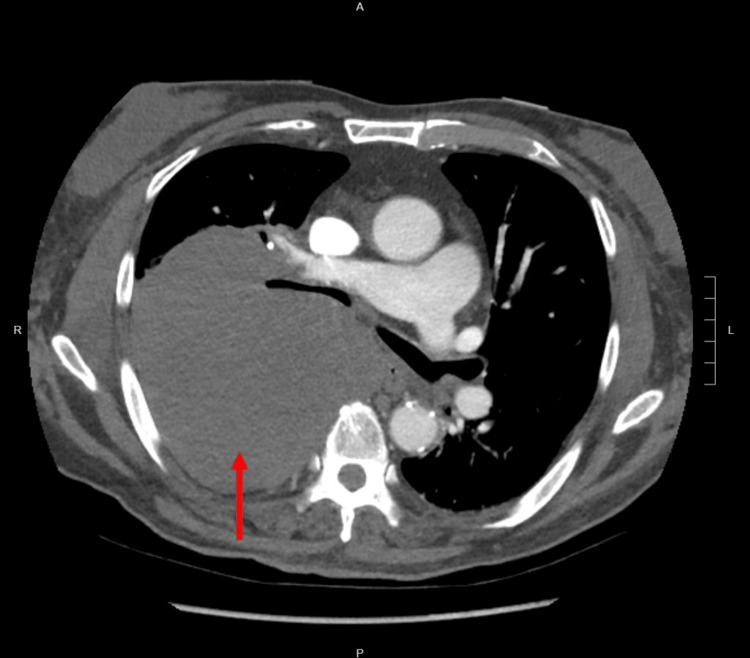
Computed tomography pulmonary angiogram identified a 12.2 cm x 12.9 cm x 16.0 cm mass occupying the majority of the right posterior hemithorax

A 69-year-old man with a history of alcohol dependence syndrome presented to the hospital with shortness of breath and malaise for one week. The patient was a smoker with more than 30 pack-years. Physical examination revealed diminished movements and breath sounds on the right hemithorax, with no signs of superior vena cava compression syndrome. Chest radiograph identified a uniform density opacity in the right upper and middle hemithorax. Computed tomography pulmonary angiogram found no evidence of pulmonary embolism, but identified a 12.2 cm x 12.9 cm x 16.0 cm non-enhancing mass occupying the majority of the right posterior hemithorax, with compression of the right main bronchus and segmental bronchi (Figure 1). Enlarged lymph nodes up to 3.9 cm in diameter were seen in the mediastinum. PET-CT detected a large necrotic right upper lobe (RUL) lung mass, with no evidence of a tumor in the mediastinum or in the testes.

CT-guided core biopsy obtained 4 cores of the right lung mass. The patient’s blood tests revealed chronic respiratory acidosis with metabolic compensation. A panel of serum tumor markers, including hCG, was tested as part of the workup for malignancies (Table 1). Measurement of hCG using the Roche Elecsys HCG+β assay (Roche Diagnostics, Basel, Switzerland), which recognizes intact hCG, nicked beta subunits (hCGβn), free beta subunits (hCGβ), and beta core fragments (hCGβcf), found an extremely elevated serum total hCG level of 24544 IU/L.

**Table 1:** Serum tumor marker results AFP: Alpha-fetoprotein; CA 19-9: Carbohydrate antigen 19-9; CA 15-3: Cancer antigen 15-3; CEA: Carcinoembryonic antigen; hCG: Human chorionic gonadotropin; PSA: Prostate-specific antigen.

Tumor marker	Result	Reference Intervals
AFP	<2 ng/mL	<9 ng/mL
CA 15-3	9.9 U/mL	<31.3 U/mL
CA 19-9	44 U/mL	<37 U/mL
CEA	35 ng/mL	<5 ng/mL
hCG	24544 IU/mL	<2 IU/L
PSA	3.5 ng/mL	<4 ng/mL

**Figure 2:**
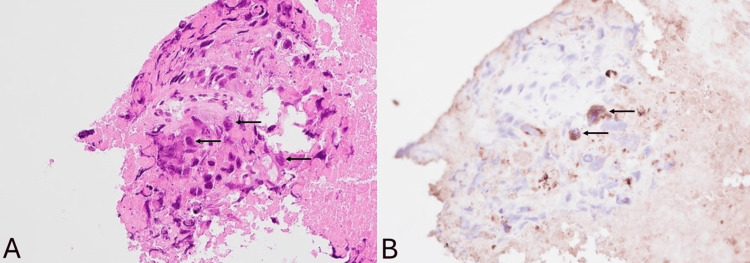
Histological examination of the lung mass specimens. (A) H&E staining showed pleomorphic tumor cells with necrosis. (B) Immunohistochemical staining showed scattered hCG-positive syncytiocytotrophoblasts. H&E: hematoxylin and eosin; hCG: human chorionic gonadotropin.

Histological examination of the right lung mass revealed tumor tissue with diffuse necrosis. The tumor cells possessed enlarged pleomorphic nuclei with irregular nuclear contour and conspicuous nucleoli (Figure 2A). Mitosis and apoptotic bodies were readily seen. Immunostaining of the tumor cells was positive for MNF-116 and patchily positive for p40, while negative for leukocyte common antigen (LCA (CD45)), S100, smooth muscle actin (SMA), thyroid transcription factor 1 (TTF-1), and synaptophysin.

It would be tempting to report the tumor as squamous cell carcinoma, which can be defined as “a poorly differentiated tumor with immunohistochemical evidence of squamous cell differentiation (positive p40 and negative TTF1)” according to the WHO classification of thoracic tumors [10]. The clinical presentation of a male smoker in his late 60s is also typical of a squamous cell carcinoma patient. Despite such factors favoring squamous cell carcinoma, the extremely elevated level of serum hCG prompted further analysis for the possibility of choriocarcinoma. Additional immunostaining revealed the tumor cells were positive for spalt-like transcription factor 4 (SALL4) and GATA-binding protein 3 (GATA3) and were focally positive for hCG (Figure 2B). With such an immunophenotype, the pleomorphic cells were essentially cytotrophoblasts and hCG-positive syncytiotrophoblasts. Hence, the diagnosis of primary pulmonary choriocarcinoma was established.

Given the very advanced disease and moribund state, the patient and his family opted for symptomatic palliative care. The patient died one month after presentation.

## Discussion

In most cases of PPC, patients typically die of disseminated disease 1 to 2 months after diagnosis [10]. PPC has been postulated to arise from retained primordial germ cells that migrate abnormally during embryogenesis, metastasis from a gonadal choriocarcinoma that regressed spontaneously, or transformation from a non-trophoblastic choriocarcinoma [4]. Trophoblastic differentiation of cells of adenocarcinoma was postulated to be the cause of combined PPC and adenocarcinoma, where reported cases found no clear boundary between the two types of carcinomas [2,7-9].

Needle biopsies of PPC may appear as squamous cell carcinoma [3-5] or poorly differentiated giant cell carcinoma [6]. One case initially appeared as squamous cell lung carcinoma on fine needle aspiration, mimicking anaplastic large cell carcinoma with giant cells on excised tumor, before reexamination of the pulmonary tumor finally confirmed the diagnosis of PPC [5]. PPCs may mimic squamous cell carcinoma as they may be positive for p63, p40, and cytokeratins 5/6 (CK5/6) on immunohistochemical examination [2]. Nevertheless, hCG staining alone is not sufficient for the diagnosis of PPC, as 54% of lung cancer cases showed hCG staining [11]. Since various histological types of lung carcinomas may express placental glycoproteins [11], immunohistochemical markers specific for choriocarcinomas, such as SALL4 and GATA3, are instrumental in the diagnosis of PPC.

Progressive elevations of β-hCG are useful in detecting choriocarcinoma lesions, though measurements of serum β-hCG are often neglected [1]. Notwithstanding, significant elevations of serum hCG levels in patients with pulmonary tumors are not specific to choriocarcinomas, as β-hCG levels of more than 10000 mIU/mL have also been reported in pulmonary squamous cell carcinomas [12,13], pulmonary adenocarcinoma [14], and pulmonary pleomorphic carcinoma [15]. In patients with extremely elevated serum β-hCG, as in this case, immunohistochemical staining for specific markers of PPC could be useful in identifying the presence of trophoblastic differentiation into PPC.

Mild serum β-hCG elevation above >5 IU/L has been reported in 12% of lung cancer patients [16], and could be seen more broadly in transitional cell carcinoma of the bladder and urinary tract, renal cell carcinoma, prostate cancer, gastrointestinal cancers, neuroendocrine tumors, breast cancer, ovarian cancers, and head and neck squamous cell carcinoma [17]. Clinical trials assessing the diagnostic and prognostic value of hCG are hindered by the lack of harmonization of serum hCG assays. One patient with an ovarian germ cell tumor had a 4-fold difference in serum hCG levels when the same sample was analyzed using different serum hCG assays [18]. The multitude of hCG variants and glycosylation patterns, in addition to the different affinity of immunoassay antibodies to the various forms of hCG, are major barriers to assay harmonization [19]. Total hCG has a higher sensitivity than hCGβ in detecting gestational trophoblastic diseases [20]. Further studies are required to verify whether the same pattern could be observed in extragonadal choriocarcinomas.

## Conclusions

PPC is a rare malignancy usually diagnosed in middle-aged male patients. Here, we report a case of PPC in a 69-year-old patient where the histological features of PPC mimicked squamous cell carcinoma. This case illustrates the diagnostic utility of serum hCG measurement and immunohistochemical staining for SALL4 and GATA3. PPCs are difficult to diagnose as PPCs may mimic or may be combined with other histological types of lung cancers. Hence, serum hCG measurement may be considered as a screening test for the detection of PPC, especially in non-small cell lung cancers. Immunohistochemical staining for SALL4 and GATA3 should be performed for patients with elevated levels of serum hCG.
